# Correlations between radiological and histological findings of bone remodelling and root resorption in a rodent cleft model

**DOI:** 10.1186/s13005-022-00338-x

**Published:** 2022-11-11

**Authors:** Stephan Christian Möhlhenrich, Kristian Kniha, Marius Heitzer, Zuzanna Magnuska, Benita Hermanns-Sachweh, Felix Gremse, Sachin Chhatwani, Frank Hölzle, Ali Modabber, Gholamreza Danesh

**Affiliations:** 1 Department of Orthodontics, University of Witten/Herdecke, Alfred-Herrhausen Str. 45, 58455 Witten, Germany; 2 Department of Oral and Maxillofacial Surgery, University Hospital of Aachen, Pauwelsstraße 30, 52074 Aachen, Germany; 3 Institute for Experimental Molecular Imaging, RWTH Aachen University, Forckenbeckstraße 55, 52074 Aachen, Germany; 4 Implant Pathology, ZBMT, Campus Melaten, Pauwelsstraße 17, 52074 Aachen, Germany

**Keywords:** Histology, Micro-computed tomography, Quantitative bone morphometry, Root resorption, Bone substitutes, Cleft animal model, Rat

## Abstract

**Background:**

The evaluation of bone remodelling and dental root resorption can be performed by histological techniques or micro-computed tomography (micro-CT). The present study aimed to evaluate the relationship between these two procedures in the context of cleft repair in a rat model.

**Methods:**

The reconstructed maxillae and the orthodontically-moved first molar of 12 rats were analysed for correlations between the histological and radiological findings retrospectively. The alveolar cleft repairs were performed using bone autografts or (human) xenografts. Four weeks after the operation, the intervention of the first molar protraction was initiated and lasted for eight weeks. The newly formed bone and the root resorption lacunae were determined via histology. In the micro-CT analysis, the average change of bone mineral density (BMD), bone volume fraction (BV/TV), trabecular thickness and trabecular separation of the jaw, as well as the volume of the root resorptions were determined. The Pearson correlation coefficient was applied to study the associations between groups.

**Results:**

Positive correlations were found only between the newly formed bone (histology) and BMD changes (micro-CT) in the autograft group (*r* = 0.812, 95% CI: 0.001 to 0.979, *p* = 0.05). The relationship of newly formed bone and BV/TV was similar but not statistically significant (*r* = 0.691, 95% CI: −0.274 to 0.963, *p* = 0.013). Regarding root resorption, no significant correlations were found.

**Conclusions:**

Due to the lack of correlation between histological and radiological findings of bone remodelling and the development of root resorptions, both methods should be combined in this cleft model in rats for a comprehensive analysis.

## Introduction

Osteoplasty is an established treatment for cleft patients. In alveolar ridge repairs, various types of bone grafts have been applied, such as autografts from the iliac crest, cranium, mandibular symphysis, tibia or rib, allografts, xenografts or synthetic bone substitutes (e.g., bioceramics, polymers or biocomposites) [1–3]. Particularly, the autologous iliac crest grafts are considered the gold standard for cleft repair due to their osteogenic, osteoinductive and osteoconductive properties [4]. Nevertheless, the bone grafting process implies operational risks and may lead to postoperative donor site morbidities, such as pain, hematoma and delayed ambulation. Furthermore, maxillofacial donor sites entail limited bone supply, indicating the demand for an additional donor site and the associated inherent susceptibility to resorption in the long term [5–10]. Therefore, different grafting materials and bone substitutes are constantly being improved to enhance clinical outcomes and limit postoperative morbidities [3, 4, 11].

In this context, we recently introduced a new alveolar cleft model in rats with complete maxillary interruption covered by the epithelial lining, which allows for orthodontic tooth movement after cleft repair [12–15]. This model permits cleft repairs using autologous bone grafts from a novel donor site, the ischial tuberosity of the hip [12, 13]. This model permits the *in vivo* radiological analysis of the bone structure of the reconstructed area and the associated tooth roots, as well as the corresponding histopathological examination after the trial. The bone graft quality of different substitutes, including autografts, and the root resorptions after cleft repair in the context of subsequent orthodontic treatment have been analysed and compared via radiology and histology methods [14, 15].

Several studies have shown that micro-computed tomography (micro-CT) measurements of bone morphology are highly consistent and accurate. Morphology assessments with micro-CT have been compared to typical two-dimensional (2D) histomorphometry measurements in both animal [16–19] and human specimens to determine their compatibility [20–24], revealing that micro-CT assessments of 2D and three-dimensional (3D) morphology correlate with 2D histomorphometry measurements. In this context, Müller et al. [21] reported high correlations and minor differences between conventional histology and microtomography analyses regarding bone volume density (BV/TV), bone surface density (BS/TV), trabecular thickness (Tb.Th) and trabecular separation (Tb.Sp).

The analysis of orthodontically induced root resorption can also be performed with micro-CT analysis [25, 26]. However, to our knowledge, there are no comparative studies on the differences between micro-CT assessments of 2D and 3D morphology and 2D histomorphometry measurements of root resorptions.

In our recently published articles [14, 15], contradictory results or discrepancies between the radiological and histological analyses were found. As for the bone microarchitecture of substitutes, bone mineral density (BMD) and BV/TV were higher in the micro-CT of the autograft than the human xenograft, while in the histological analysis, the most persistent grafting material was the human xenograft, which also led to the highest percentage of new bone formation [15]. The largest root resorption was detected at the mesial root after orthodontic tooth movement of the autograft with the radiological analysis, followed by the human xenograft [14]. Based on these findings, the previously documented correlation between histological and radiological measurements in different tissues does not seem to exist in this model.

Therefore, this follow-up investigation aimed to evaluate the correlation between histological findings by toluidine blue staining and radiological findings in micro-CT analysis in the case of root resorptions and bone morphology changes after cleft repair. Particularly, we determined whether new bone formation can be deduced from the radiological analysis of bone morphology.

## Materials and methods

Detailed information about the study protocol and the procedures was recently published [12, 13]. The *a priori* sample size calculation was performed applying a one-way ANOVA considering the data in Ru et al. [27].

The animal trials were authorised by the Governmental Animal Care and Use Committee (Reference No.: 81–02.04.2018.A342; Landesamt für Natur, Umwelt und Verbraucherschutz Recklinghausen, Nordrhein-Westfalen, Germany; date: January 11, 2019) and were conducted in agreement with the German Animal Welfare Law (Tierschutzgesetz, TSchG) and the European Union Directive 2010/63/EU. The study was performed according to the ARRIVE Guidelines [26] and the Guide for the Care and Use of Laboratory Animals.

In this follow-up investigation, the rats were retrospectively analysed after alveolar cleft repair with either autograft or human xenograft (*N* = 6 per group). In one group, the cleft repair was performed with autologous bone from the hip, while in the other group, human xenogeneic bone substitute (maxgraft, Botiss Biomaterials, Zossen, Germany) was used.

**Fig. 1:**
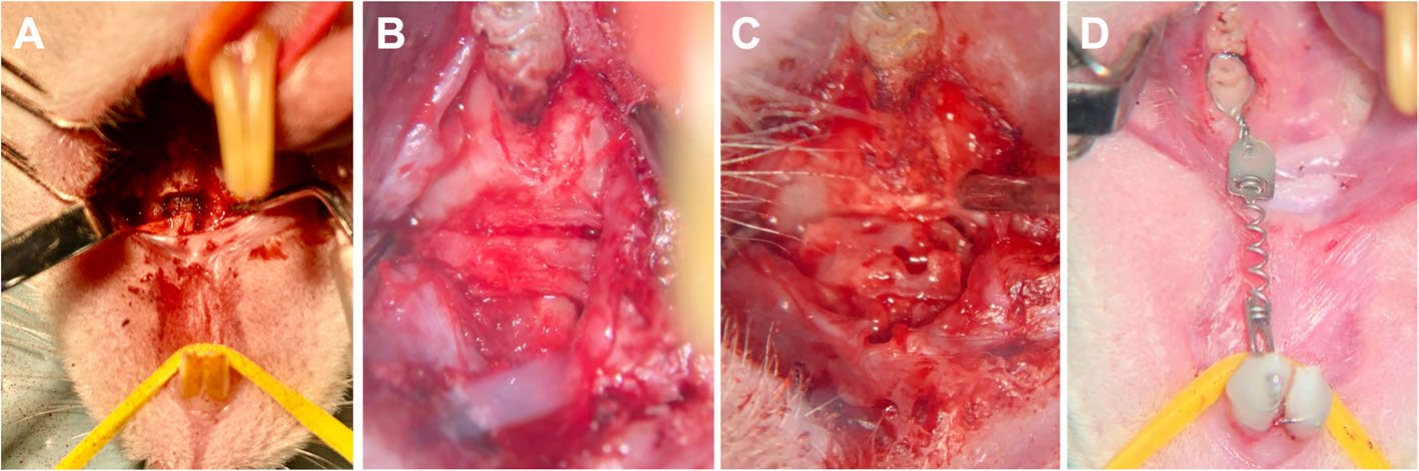
View of the surgical site on the left maxilla and palate. Top: tongue base; bottom: mouth tip. **A** Preparation of the artificial alveolar cleft in the front of the first molar on the left side of the rat’s maxilla with an ultrasonic device. **B** Maxillary cleft repair was performed with an autograft from the ischial tuberosity of the hip or (**C**) a human xenograft. **D** Orthodontic appliance based on a 0.14 N nickel/titanium closed coil tension spring fixed between the first molar and the incisors

At the time of the artificial maxillary cleft creation, the rats were 8 weeks old and had a mean average weight of 465 ± 34 g, while after another 4 weeks the cleft repair was performed. Finally, four weeks after the maxillary reconstruction, the orthodontic tooth movement intervention started and lasted for 8 weeks (Fig. 1A–C). At the end of the experimental procedures, the animals were 16 weeks old with an average body weight of 542 ± 32 g. Subsequently, the animals were euthanised by cervical dislocation under general anaesthesia.

Cleft creation, maxillary reconstruction and orthodontic device placement were performed under general anaesthesia with a cocktail of ketamine (80–100 mg/kg, i.p.), medetomidine hydrochloride (0.15–0.25 mg/kg, i.p.) and buprenorphine (0.03–0.05 mg/kg, s.c.). Antibiotic administration (cefuroxime: 15 mg/kg, s.c.) started after the operation at a 24-hour interval for seven consecutive days. Atipamezole hydrochloride (0.75 mg/kg, i.p.) was administered to support the recovery process, and buprenorphine (0.03–0.05 mg/kg, s.c.) was given for maximum 5 days when necessary.

### Micro-computed tomography (Mirco-CT) analysis

The required radiological examinations were performed *in vivo* using a μCT system (U-CT OI, MILabs, Utrecht, the Netherlands) at three time points: immediately after the jaw reconstruction (T0), 4 weeks after cleft repair and before the initiation of the orthodontic tooth movement (T1), and 12 weeks after cleft repair or 8 weeks of orthodontic tooth movement (T2).

The radiology parameters were the following: ultra-focus magnification and rotation of 360° at an increment of 0.75° with 0.3 s/degree. The data were reconstructed at an isotropic voxel size of 40 μm. The Micro-CT data were down-sampled to a voxel size of 80 μm. The images of cross-sectional slices were rendered to 3D iso-surfaces. For the analysis of the reconstructed maxillae and the root resorption of the first molars, both regions were segmented in micro-CT images using all the anatomical planes.

**Fig. 2:**
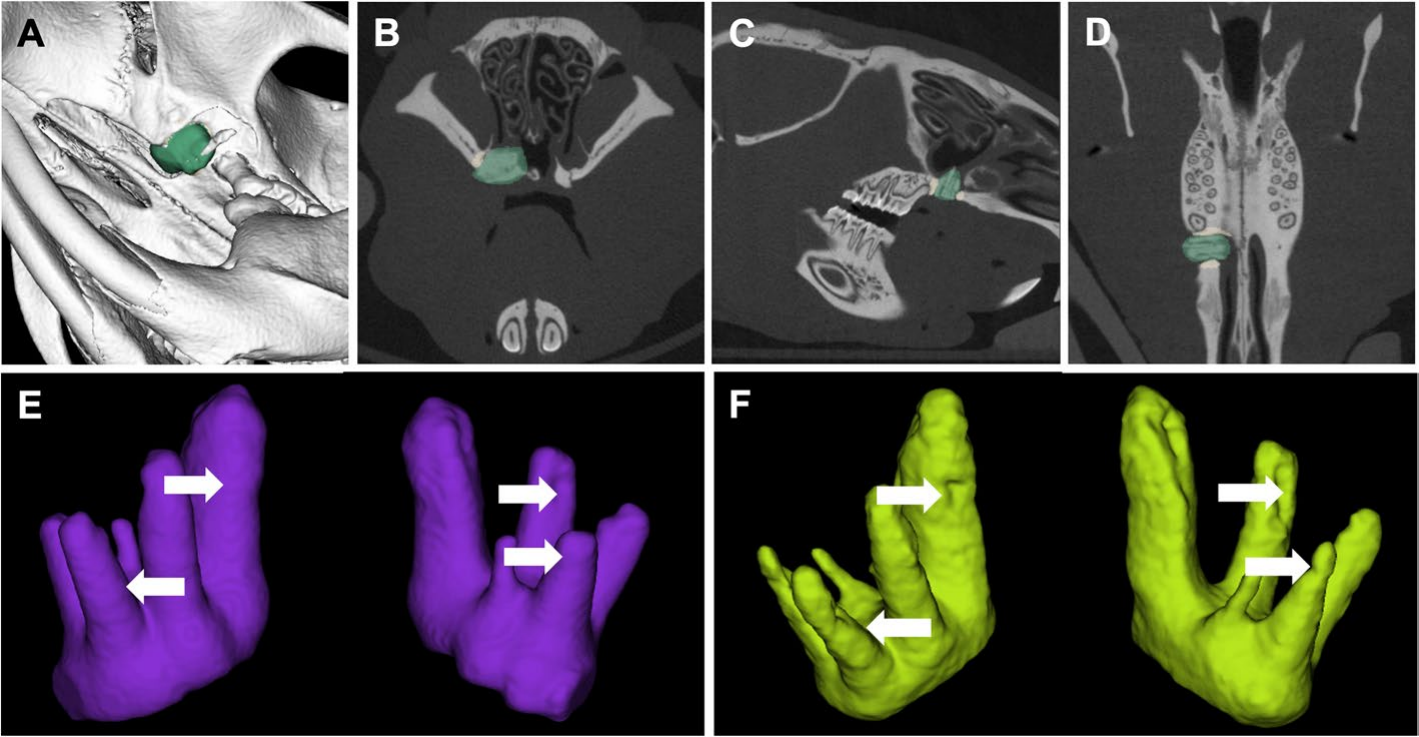
Radiological imaging after cleft repair. **A** The autologous bone (in green) in three-dimensional (3D) micro-computed tomography (micro-CT) volume rendering (**B**) in the transverse, (**C**) coronal and (**D**) sagittal planes. (**E**) Three-dimensional reconstruction of the extracted teeth before (in purple) and (**F**) after (in yellow) the orthodontic treatment. The white arrows point to root resorption signs

For the bone analysis, a coat with a fixed 10-voxel thickness was calculated around the segment using the morphological operation [28]. Then, the bone tissue was segmented within the coat’s volume via thresholding. The reconstructed maxilla and the surrounding bone were then analysed for BMD, BV/TV, Tb.Th and Tb.Sp (Fig. 2A–D). Radiological changes (Δ = T2-T0) within the reconstructed part of the maxilla were defined by the difference among these measurements.

For the root analysis, all roots were individually delineated, and their volumes were calculated for all three measurements (T0–T2). The root resorption was analysed by subtracting the root volume at T2 from the root volume at T1 (Fig. 2E–H).

### Histomorphometry analysis

After resection of the affected part of the maxilla including the orthodontically moved first molar, the samples were stored in 4% formalin (Otto Fischar GmbH & Co. KG, Saarbrücken, Germany), followed by decalcification in a 20-fold volume of ethylenediaminetetraacetic acid (EDTA, MolDecalcifer, Menarini, Florence, Italy) for 4 weeks at 37 °C. Afterwards, the samples were deposited in 5% sucrose/phosphate-buffered saline for 24 h, followed by shock freezing in liquid nitrogen and finally embedded (TissueTek, Sakura, Alphen, Netherlands).

Subsequently, longitudinal sections through the tooth and the surrounding hard and soft tissue or cross-sections from the area immediately in front of the first molar (all sections were 7 μm thick) were collected and fixed on super frost slides for drying. Then, the samples were submerged in acetone for 10 min and stained with toluidine blue, according to a standard protocol. The specimens were observed under digital microscopy with software support (OLYMPUS digital microscope DSX-1000, Olympus Hamburg, Germany).

**Fig. 3:**
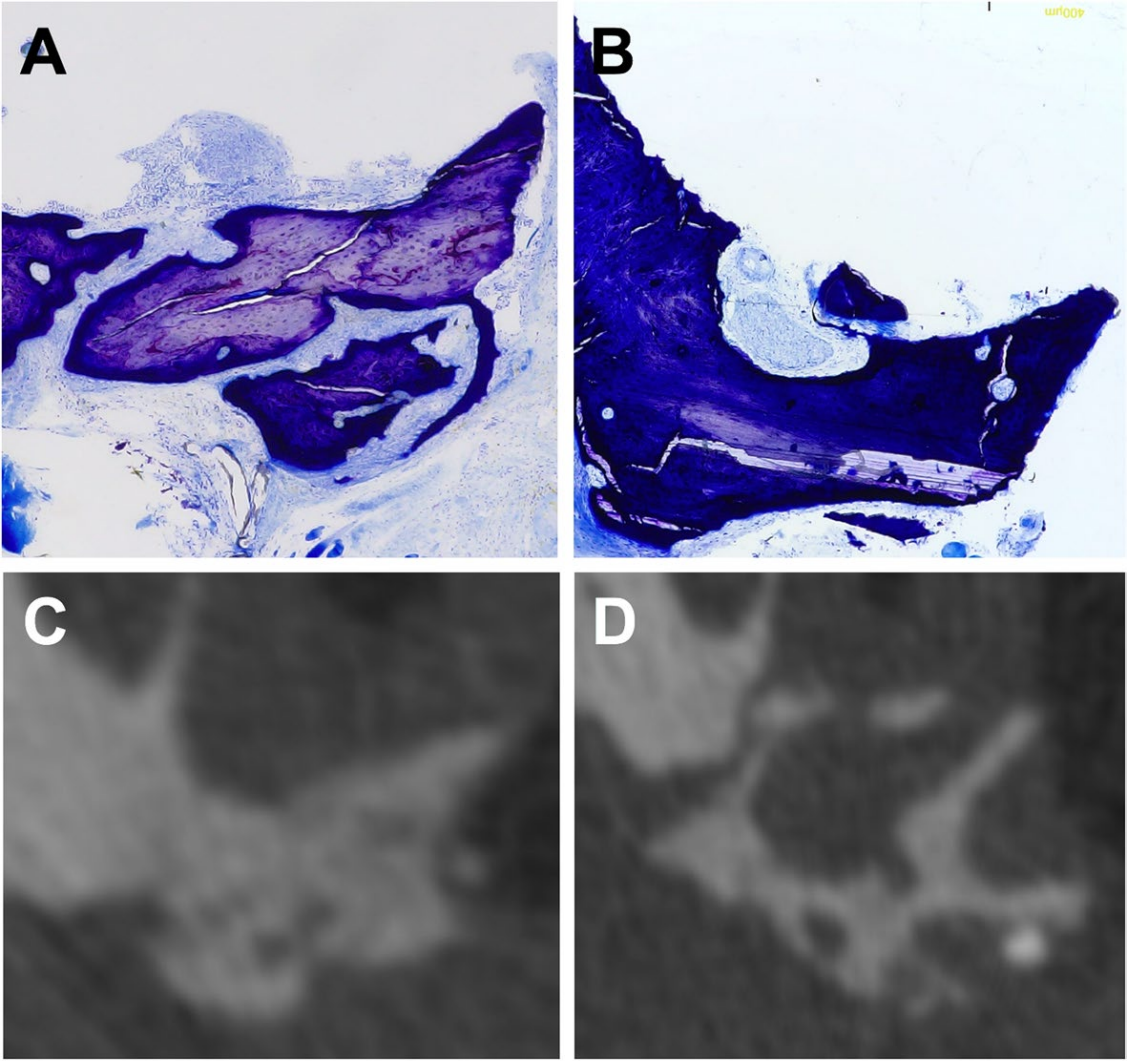
Toluidine blue staining of the reconstructed jaw after cleft repair and orthodontic tooth movement. Microscopy imaging of (**A**) the autologous bone and (**B**) the xenogeneic/human bone. Representative radiological slices on the transverse plane for (**C**) the autologous and (**D**) the xenogeneic/human bone

**Fig. 4:**
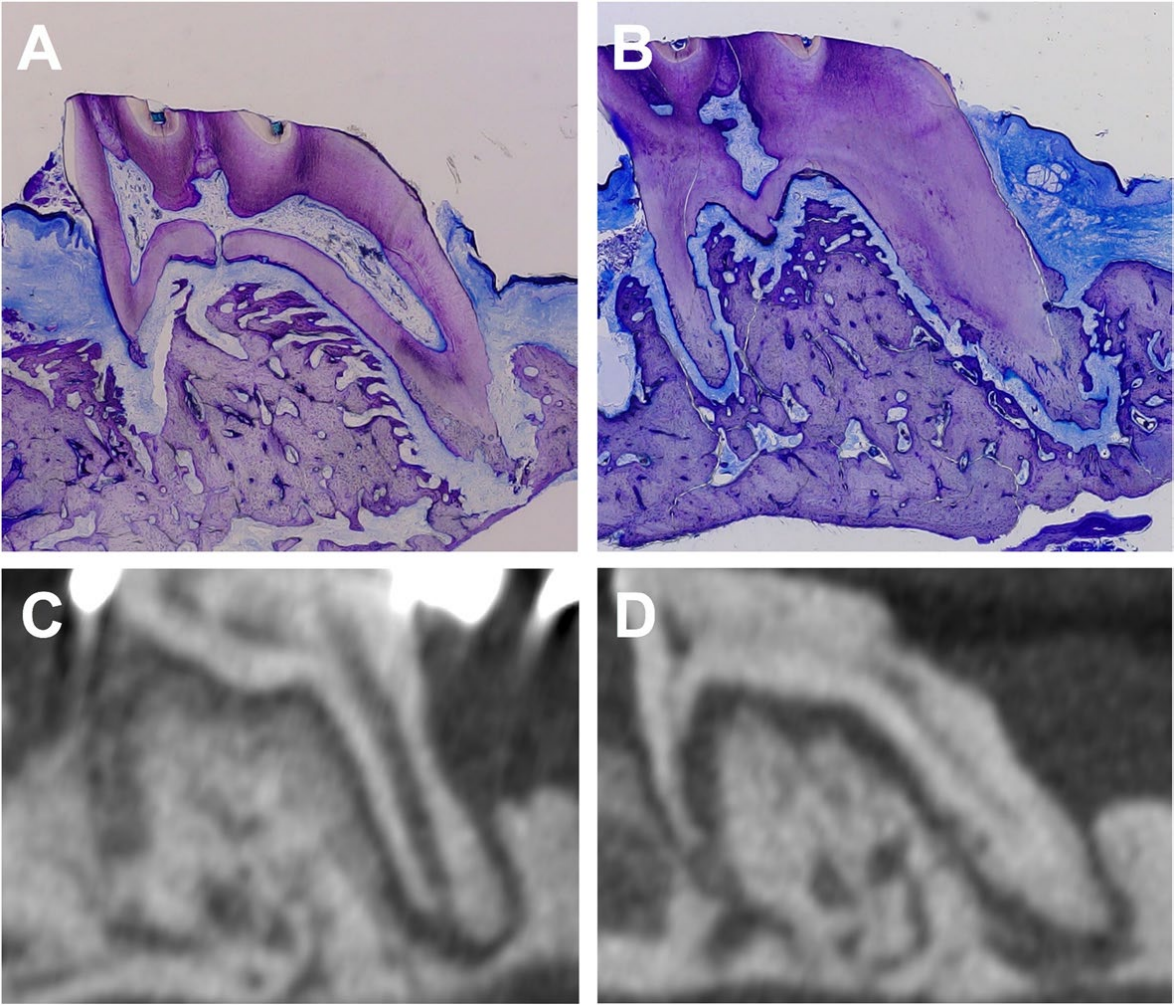
Toluidine blue staining of longitudinal sections of the first molar and the surrounding hard and soft tissue after cleft repair and orthodontic tooth movement. **A** The autologous and (**B**) xenogeneic/human bones. Representative radiological slices on the transverse plane for (**C**) the autologous and (**D**) the xenogeneic/human bone

The region of the augmented bone, the newly formed bone, and the interior and exterior of the augmented substitutes were observed to evaluate the osseous build-up or the bone substitutes that were still present (Fig. 3). The amount of root resorption was defined as the area between the intact parts of the root surfaces (Fig. 4).

### Statistical analysis

The Shapiro-Wilk test was applied to confirm the normal distribution of the data. Statistical comparisons between the groups were performed with the unpaired nonparametric Mann-Whitney test with compared ranks. The correlations between the radiological (BMD, BV/TV, Tb.Sp and Tb.Th) and the histological measurements to determine the newly formed bone (mm^2^) and the resorption lacunae (mm^2^) were analysed with the Pearson correlation coefficient (*r*). The latter was also used to study the correlation between the radiological and histological determination of root resorptions. All analyses were performed on Prism (version 8, GraphPad Software Inc., La Jolla, CA, USA). The level of significance was set at *p* ≤ 0.05. All results are expressed as mean ± standard deviation (SD).

## Results

**Fig. 5:**
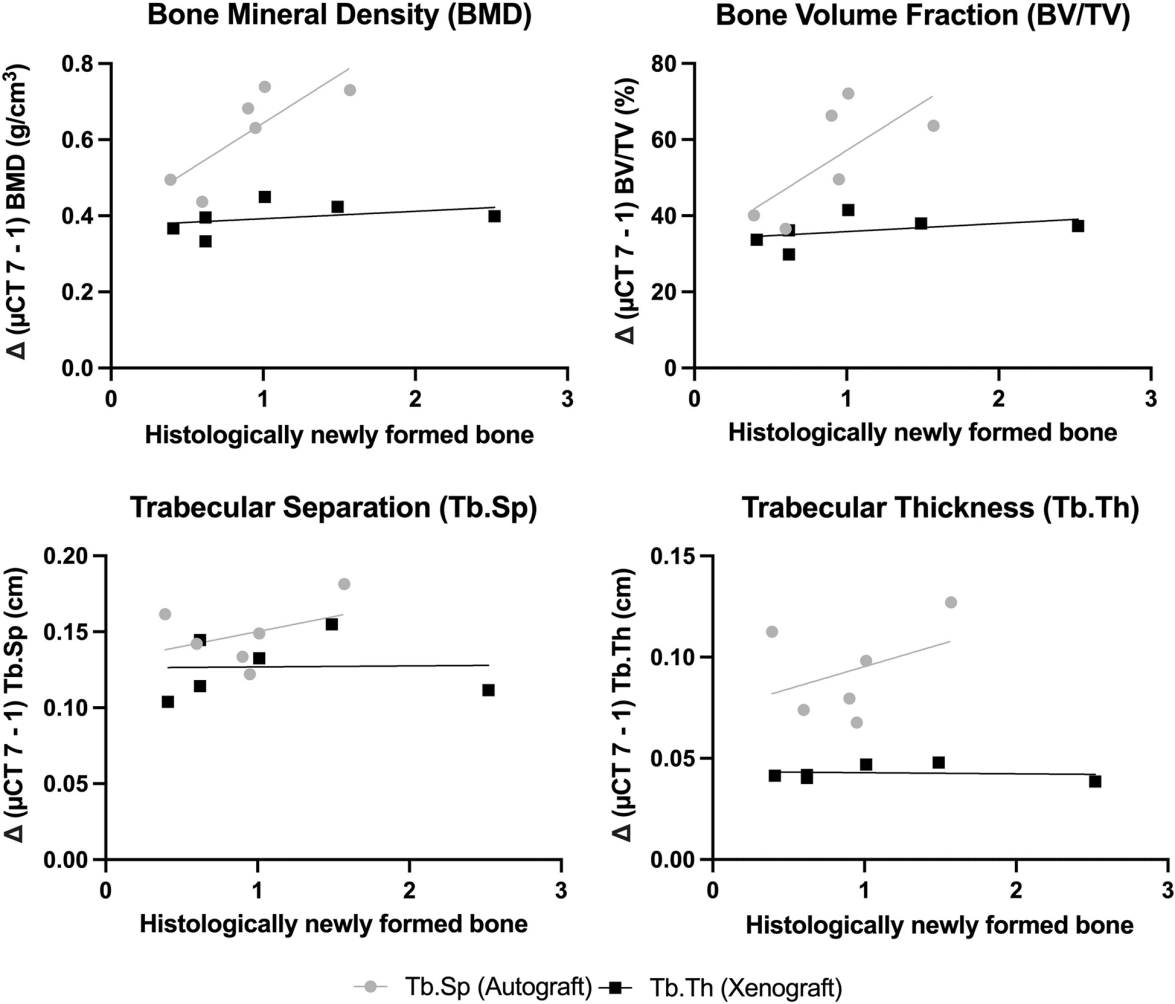
Scatter plot demonstrates the lack of correlation between the histology results of newly formed bones and the radiology results of the changes of BMD, BV/TV, Tb.Sp and Tb.Th for both bone substitutes using Pearson r correlation test, *p* < 0.005

**Fig. 6:**
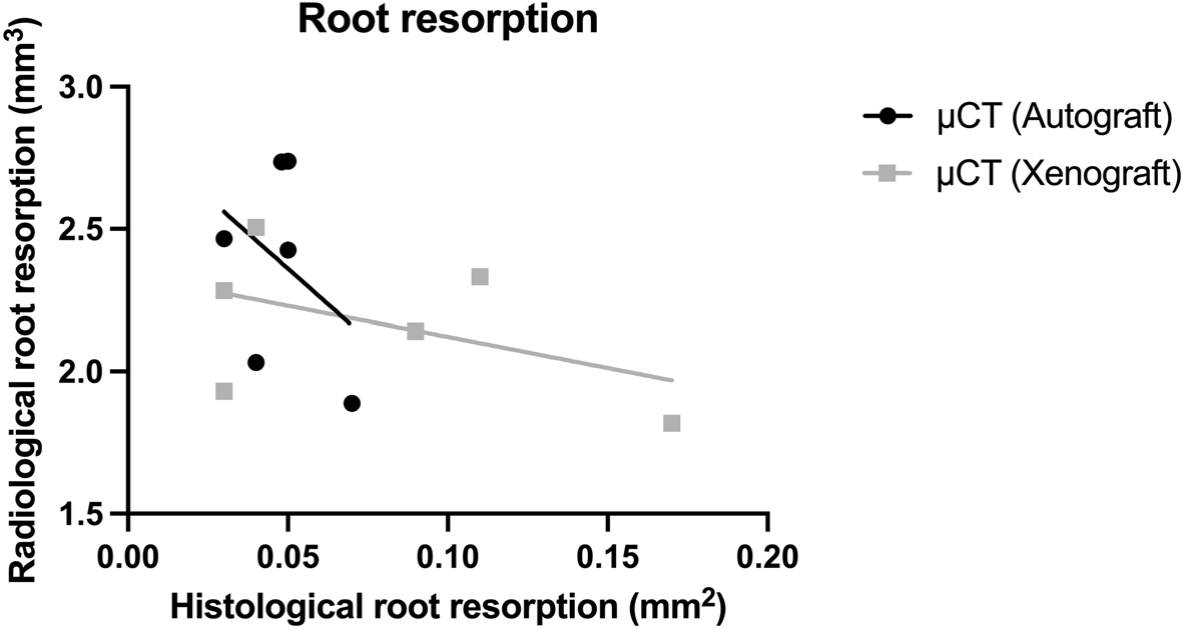
Scatter plot demonstrates the lack of correlation (Pearson test) between the histology and radiology results for root resorptions for both bone substitutes using Pearson r correlation test,, *p* < 0.005

The results of the histological analysis (persistent grafting material and new bone formation measured in mm^2^) and the radiological measurements (changes in BMD, BV/TV, Tb.Th and Tb.Sp) 12 weeks after the cleft repair, including the 8 weeks of tooth movement, are shown in Table 1. The mean values of root resorptions are demonstrated in Table 2. Table 3 presents the results of the correlation analysis. Figure 5 shows the overall relationship between the histological findings of newly formed bones and the radiological measurements of morphometric bone changes. Figure 6 presents the relationship between the histological and radiological measurements of root resorptions.

**Table 1:** Numerical results from the statistical analysis

		Autograft			Human Xenograft			***P***-Value
		Mean ± SD	Min	Max	Mean ± SD	Min	Max	
**Histological analysis**	Grafting material (mm^2^)	0.90 ± 0.40	0.39	1.57	1.11 ± 0.79	0.41	2.52	0.74
	New bone formation (mm^2^)	0.51 ± 0.29	0.08	0.95	0.89 ± 0.84	0.28	2.52	0.69
**Radiological analysis**	Δ BMD (g/cm^3^)	0.078 ± 0.139	- 0.158	0.228	- 0.031 ± 0.013	−0.049	−0.017	0.07
	Δ BV/TV (%)	- 0.183 ± 15.6	- 25.10	17.5	- 5.48 ± 1.48	−7.35	−3.52	0.39
	Δ Tb.Th (cm)	0.031 ± 0.024	0.007	0.059	- 0.003 ± 0.001	−0.004	−0.003	0.002*
	Δ Tb.Sp (cm)	0.039 ± 0.022	0.010	0.060	0.014 ± 0.019	−0.018	0.038	0.065

The amount of persistent grafting material (mm2) and new bone formation (mm2) after the 12-week cleft repair healing period (T0–T2) as evaluated by histology. The changes (Δ = Mirco-CT 2–0) in bone mineral density (BMD; g/cm3), bone volume fraction (BV/TV; %), trabecular thickness (Tb.Th; cm) and trabecular separation (Tb.Sp; cm) as evaluated by radiology. The data are presented as mean, minimum and maximum values ± standard deviation (SD)

**Table 2:** Mesial root resorption as measured with histology (mm2) and radiology (mm3) after 8 weeks of tooth movement (T1–T2)

		Autograft			Human Xenograft			***P***-values
		Mean ± SD	Min	Max	Mean ± SD	Min	Max	
**Histological analysis**	(mm^2^)	0.05 ± 0.01	0.03	0.07	0.08 ± 0.05	0.03	0.17	0.72
**Radiological analysis**	(mm^3^)	2.38 ± 0.35	1.89	2.74	2.17 ± 0.26	1.82	2.51	0.31

The data are presented as mean, minimum and maximum values ± standard deviation (SD)

**Table 3:** Correlations between the radiology (Δ = μCT 2–0 in BMD, BV/TV, Tb.Sp and Tb.Th) and the histology measurements for new bone formation and the development of root resorptions according to the applied bone substitutes

		Number of pairs (N)	Pearson Correlation		
			Rank Correlation (r)	95% confidence interval	***P***-value
**Bone substitute**	**Δ (μCT7–1)**				
Autograft	BMD	6	0.812	0.001 to 0.979	0.05*
	BV/TV	6	0.691	−0.274 to 0.963	0.13
	Tb.Sp	6	0.377	−0.626 to 0.910	0.46
	Tb.Th	6	0.378	−0.626 to 0.910	0.46
Human Xenograft	BMD	6	0.373	−0.629 to 0.909	0.47
	BV/TV	6	0.422	−0.593 to 0.919	0.40
	Tb.Sp	6	0.0259	−0.803 to 0.820	0.96
	Tb.Th	6	−0.115	−0.847 to 0.768	0.83
**Root resorption**					
Autograft		6	−0.373	−0.909 to 0.629	0.139
Human Xenograft		6	−0.473	−0.928 to 0.550	0.223

* statistically significant

As for the bone morphometric changes, neither the histological nor the radiological analysis showed statistically significant differences between the autograft and the human xenograft groups, except for Tb.Th (0.031 ± 0.024 *vs.* -0.003 ± 0.001, *p* = 0.002). Additionally, no statistically significant differences were found between the autograft and the xenograft groups in the case of the root resorptions, regardless of the analysis method.

The only positive relationship was found between the histological findings of newly formed bone and the radiological measurements of BMD changes in the autograft group (*r* = 0.812, 95% CI: 0.001 to 0.979, *p* = 0.05). The comparison between the amount of newly formed bone and BV/TV is of similar magnitude but not statistically significant (*r* = 0.691, CI [−0.274 to 0.963], *p* = 0.13). Regarding root resorptions, no significant correlations were found.

## Discussion

A large number of preclinical animal studies on cleft repair have examined materials alternative to autologous bone. The bone substitutes include allografts, xenografts and synthetic substitutes (e.g., bioceramics, polymers, or biocomposites) [1–3]. To improve clinical outcomes and decrease postoperative morbidity, bone substitutes have been tested [3, 4, 11]. In this context, various artificial cleft models in rats have been introduced to analyse the bone remodelling processes and the development of root resorptions through evaluation with traditional histological techniques or radiological methods such as Micro-CT [27, 29–36].

Generally, histological methods for morphological analyses require considerable preparation of the samples, i.e., embedding in methylmethacrylate and, subsequently, sectioning. Even though the method offers high longitudinal resolution and image contrast, it is labour-intensive and time-consuming. Additionally, it deteriorates the samples, preventing repeated measurements of the same specimen at different time points.

To overcome these limitations, a variety of 3D visualisation techniques have become popular [37]. Micro-CT is an alternative for 3D imaging and quantification of bone structures. Müller et al. [21] compared histology (2D) and micro-CT (3D) regarding BV/TV, BS/TV, Tb.Th and Tb.Sp, revealing significant correlations between the two methods for all the morphometric parameters. They concluded that the non-destructive, fast and precise radiological analysis allows the measurement of bone structures without biopsies of small bone samples [21].

Micro-CT has also been used in experimental research with laboratory animals in the field of cleft treatment [27, 36, 38, 39]. Recently, we published the results of our investigations on different graft materials used in alveolar cleft repair, the subsequent bone healing process and the development of dental root resorptions in the context of orthodontic tooth movement in a rodent model [14, 15]. In the autologous bone graft group, BMD increased from 0.54 ± 0.05 g/cm^3^ to 0.62 ± 0.11 g/cm^3^, while in the xenograft group, the values remained unchanged (from 0.43 ± 0.04 g/cm^3^ to 0.40 ± 0.04 g/cm^3^) [15]. As for BV/TV, it remained unchanged in the autografts group (from 54.89% ± 5.07 to 54.71% ± 14.74%), while in the human xenografts group, it decreased (from 41.55% ± 5.27 to 36.07% ± 3.99%). In contrast, the histological findings showed an increase in the newly formed bone in both groups (autograft: 0.89 ± 0.29 mm^2^; xenograft: 0.52 ± 0.84 mm^2^), which was also reflected in the distribution of the newly formed bone on the persistent bone substitute (autograft: 79.45%; xenograft: 62.18%) [15]. The radiological examination demonstrated an increase in the mesial root resorption in both the autologous (2.38 ± 0.35 mm^3^) and the xenogeneic groups (2.17 ± 0.26 mm^3^), while in the histological analysis, resorption in the autologous group was 0.048 ± 0.015 mm^2^ and in the xenogeneic group 0.078 ± 0.05 mm^2^ [14]. The bone changes measured with micro-CT were partially in contrast to the histological measurements of new bone formation.

Based on the correlations between histological and radiological findings in other medical fields [21, 23, 40, 41], we investigated this relationship by studying bone repair processes and root resorptions in our rodent cleft model to determine whether both analytical procedures are necessary for the assessment of structural changes or micro-CT is sufficient. Apart from reproducing our previous findings [14, 15], Tb.Th and Tb.Sp changes were now also included in the present follow-up investigation since they represent a feature of histological analysis of the new bone formation.

Concerning the remodelling processes, weak relationships between both analysis methods were found, with the only significant correlation being that of newly formed bones (histology) and BMD changes (radiology) in the autograft group (*r* = 0.812, CI [0.001 to 0.979], *p* = 0.05). Therefore, in this experimental model, BMD, BV/TV, Tb.Th and Tb.Sp cannot be used to confirm new bone formation. Likewise, no correlations were found between the 2D root resorptions and the 3D resorption lacunae. Regarding the significantly larger total amount of root resorption in the radiological 3D imaging and the lack of a correlation with histological 2D slices, the use of histology techniques should be considered. The type of bone substitute also has a minor role in X.

Because of the specific study design, comparisons between data from the current literature and data from the present study are impossible. Micro-CT is an established method for bone analysis in different fields of medicine. Recently, Pichone et al. [41] assessed trabecular and cortical parameters using histomorphometry and micro-CT of the iliac crest bone core in haemodialysis patients, finding a moderate correlation between the techniques in the trabecular bone volume. In different conditions such as osteoporosis, hypoparathyroidism and primary hyperparathyroidism, positive correlations between the two techniques have been documented [21, 23, 40]. However, in patients with ESRD or renal osteodystrophy, no significant correlations were observed [42, 43]. Pereira et al. [44] investigated paediatric patients with renal osteomalacia, where BV/TV was higher in histomorphometry than in micro-CT, and the osteoid accumulation in histomorphometry negatively correlated with the trabecular density observed in Micro-CT.

## Conclusions

Due the missing correlation between the histolgical and radioloigical findings, a detailed and inclusive analysis needs further both kinds of preparations. However, it should be noted that in the present study the sample size was small, possibly influencing the statistical analysis.

## Data Availability

The data supporting the findings of this research can be obtained directly from the corresponding author.
